# The Humanistic Burden of Type 1 Diabetes Mellitus in Europe: Examining Health Outcomes and the Role of Complications

**DOI:** 10.1371/journal.pone.0164977

**Published:** 2016-11-03

**Authors:** Anna Rydén, Elisabeth Sörstadius, Klas Bergenheim, Alexandru Romanovschi, Fredrik Thorén, Edward A. Witt, Catarina Sternhufvud

**Affiliations:** 1 AstraZeneca, Mölndal, Sweden; 2 AstraZeneca, Mississauga, Canada; 3 Kantar Health, Princeton, NJ, United States of America; Baylor College of Medicine, UNITED STATES

## Abstract

**Aims:**

Diagnoses of Type 1 Diabetes Mellitus (T1DM) in Europe appear to be on the rise. Therefore it is imperative that researchers understand the potential impact that increases in prevalence could have on the affected individuals as well as on society as a whole. Accordingly this study examined the humanistic and economic burden of T1DM in patients relative to those without the condition across a number of health outcomes including health status, work productivity loss, activity impairment, and healthcare resource use.

**Methods:**

Survey data from a large, representative sample of EU adults (The EU National Health and Wellness Survey) were examined.

**Results:**

Results suggest that overall burden is higher for those diagnosed with T1DM than respondents without diabetes and that burden increases as complications associated with T1DM increase.

**Conclusions:**

Taken together, these results suggest that treatment strategies for T1DM should balance clinical, humanistic, and economic burden and patients should be educated on the role of complications in disease outcomes.

## Introduction

Diabetes is a metabolic disease characterized by hyperglycemia arising from defects in insulin secretion, insulin action, or both.[1] Globally, the incidence and prevalence of T1DM, characterized by defects in insulin secretion, vary substantially.[2] However, T1DM is more common in certain European countries (in particular, Finland and the Italian province of Sardinia) than in other parts of the world and its incidence in European countries is on the rise.[3] In Finland, Germany, and Norway, annual increases in T1DM incidence of 2.4%, 2.6%, and 3.3%, respectively, have been reported.[3–5] The most substantial increases have been noted in children younger than 5 years of age.[6,7]

T1DM confers the risk of an array of vascular and nerve complications. Poor glycemic control in T1DM is related to long-term damage, dysfunction, and failure of different organs, especially the eyes, kidneys, nerves, heart, and blood vessels.[1] However, intensive diabetes therapy aimed at achieving near normoglycemia reduces the risk of these microvascular (e.g., retinopathy, nephropathy) and neurologic complications of T1DM.[8] Cardiovascular disease has become a more common macrovasular complication as T1DM patients live longer.[9] In fact, T1DM patients show a ten-fold increase in risk of cardiovascular events (e.g., myocardial infarction, stroke, angina, and the need for coronary artery revascularization) relative to age-matched control subjects.[10] The Pittsburgh Epidemiology of Diabetes Complications study in T1DM reported cardiovascular events in adult patients < 40 years of age to be 1% per year. This estimate was three times higher in individuals > 55 years of age.[11] Moreover, T1DM patients have poorer outcomes than patients without diabetes after an acute coronary event.[12] Finally, T1DM may affect age of onset for skeletal disorders. In a study of prevalence of lumbar spine and femoral neck osteoporosis in German outpatients, prevalence of low bone mineral density and fractures was similar in patients with T1DM and type 2 diabetes mellitus (T2DM), but T1DM patients were about 20 years younger than T2DM patients.[13]

Complications of T1DM have been shown to impact the disease burden on European patients. For example, UK and German patients with diabetic foot complications have reported vastly reduced health-related quality of life (HRQL), especially among those requiring amputations and with intractable or recurrent ulcers.[14] A systematic review of data from European patients suffering from painful diabetic peripheral neuropathy (PDPN) reported the patients to be limited in their general functioning and their ability to sleep, experiencing increased rates of anxiety and depressive symptomatology, as well as lowered HRQL.[15]

Regarding data on T1DM-related morbidity and mortality, in a nation-wide study of the Scottish Care Information-Diabetes Collaboration database, T1DM was associated with higher CVD and death rates than the population without diabetes.[16] A meta-analysis of T1DM patients from Europe, North America, Australia, and Asia indicate that female patients are particularly at risk, with a roughly 40% greater excess risk of all-cause mortality, and twice the excess risk of fatal and nonfatal vascular events, compared to men with T1DM.[2]

Position statements and clinical guidelines–such as the joint statement including the European Diabetes Working Party for Older People (EDWPOP), and clinical guidelines from the European Society of Cardiology–call for improved T1DM control to attenuate its complications and the subsequent humanistic burden.[17,18] In order to advance these initiatives and evaluate their continued progress, data are needed to update and quantify the current impact of T1DM and its complications in European countries. The purpose of this study was to compare outcomes (HRQL, work productivity, and medical resource use) among a large sample of European individuals with a self-reported diagnosis of T1DM (*N* = 3,686) with control subjects with no diagnosis. In addition it examined the impact that T1DM complications (e.g., retinopathy, ulcers, neuropathic pain) may have on the burden experienced among T1DM patients.

## Material and Methods

### Sample

The sample for this study was drawn from the 2013 EU National Health and Wellness Survey (NHWS; N = 62,000). The NHWS is a cross-sectional general health survey of adults. The EU NHWS is fielded in five countries: UK, France, Germany, Spain, and Italy. NHWS respondents are recruited from an Internet panel using a random stratified sampling framework in order to ensure the demographic composition (with respect to age and sex) is identical to that of the adult population in each country based on governmental statistics. The study was approved by the Essex Institutional Review Board in Lebanon, New Jersey, USA and all data were anonymized prior to analysis. Written informed consent was given by participants for their information to be used in this study but no patient medical records were shared or used.

### Measures

#### Demographic and Health Characteristics

The following demographic and health characteristics were collected for all respondents–age, sex, marital status, education, income, BMI, smoking status, alcohol status, and exercise status. Additionally the presence of a number of comorbidities (diagnoses) was assessed. These diagnoses were then used to score the Charlson Comorbidity Index (CCI). The CCI is a weighted index of comorbidities known to be predictive of mortality. Each endorsed comorbidity from the CCI is given a score between 1 and 6 and then these scores are summed into an index with higher scores indicating greater comorbidity burden. The CCI includes the following comorbidities: HIV/AIDS, metastatic tumor, lymphoma, leukemia, any tumor, moderate/severe renal disease, hemiplegia, type 2 diabetes, mild liver disease, ulcer disease, connective tissue disease, chronic pulmonary disease, dementia, cerebrovascular disease, peripheral vascular disease, myocardial infarction, congestive heart failure, and type 2 diabetes with end organ damage. The greater the total index score, the greater the comorbid burden on the respondent.

#### T1DM Status

Participants were provided with a list of medical conditions including the option “*Diabetes (Type 1 or Type 2)*” and asked “*Which of the following conditions have you ever experienced*?*”*. If patients selected “*Diabetes (Type 1 or Type 2)*” at this step they were then asked the question “*Has your [Diabetes (Type 1 or Type 2)] been diagnosed by a physician*?*”* If the participant selected a response of “*Yes*” for this question he or she qualified for the Diabetes condition series (a series of questions specific to Type 1 and Type 2 diabetes). The first question of this series was “*You indicated that you have experienced diabetes*. *What kind of diabetes do you have*?” and have the option of selecting “*Type1 or juvenile diabetes*” OR “*Type 2 or adult onset diabetes*”. For all analyses, those who selected “*Type1 or juvenile diabetes*” were coded as “T1DM” (n = 402). Those respondents who did not indicate either response were labeled as “*No diabetes*” (n = 57,912), and those who answered “*Type 2 or adult onset diabetes*” (n = 3,686) to the diagnosis question were excluded from all analyses.

#### Number of Complications

T1DM patients (n = 402) were further broken down into analysis groups by counting the number of complications they selected from the following list: Macular Edema/Diabetic Retinopathy, Neuropathic Pain, Kidney Disease, End Organ Damage, Foot or Leg Ulcer. In order to keep analysis groups of a reasonable size, the final groupings were no complications (“None”; n = 270), one complication (“One”; n = 76), and two or more complications (“Two or more”; n = 56).

#### Health status

Was measured via the SF-36v2[19] standard. This instrument reports on eight health concepts (physical functioning, role physical, bodily pain, general health, vitality, social functioning, role emotional, and mental health). From these two summary scores are also calculated: physical component summary (PCS) and mental component summary (MCS) scores. Both the eight domain scores and the two summary scores are normed by transforming the raw scores for the items to a mean of 50 and a standard deviation of 10 for the population. Higher scores indicate better health status.

#### Work Productivity and Activity Impairment

Was assessed using the Work Productivity and Activity Impairment (WPAI) questionnaire, a 6-item validated instrument that consists of four metrics: absenteeism (the percentage of work time missed because of one's health in the past seven days), presenteeism (the percentage of impairment experienced while at work in the past seven days because of one's health), overall work productivity loss (an overall impairment estimate that is a combination of absenteeism and presenteeism), and activity impairment (the percentage of impairment in daily activities because of one's health in the past seven days).[20] Only respondents who reported being full-time or part-time employed provided data for absenteeism, presenteeism, and overall work impairment. All respondents provided data for activity impairment.

#### Healthcare Utilization

Healthcare utilization was defined by the number of traditional healthcare provider visits (*“How many visits did you make to the following traditional healthcare provider(s) in the past six months*?*”*), the number of ER visits ("*how many times have you been to the emergency room for your own medical condition in the past six months*?"), and the number of hospitalizations ("*how many times have you been hospitalized for your own medical condition in the past six months*?") reported in the past six months. The phrasing “own medical condition” is used to ensure that trips to accompany a friend or relative for their medical issues were not included in the calculation. The phrasing is intentionally vague so that all medical conditions are included.

### Statistical Analyses

Differences between groups (T1DM vs. No Diabetes or None vs. One vs. Two or More complications) were calculated using means and standard deviations for continuous variables and frequencies and percentages for categorical variables. These differences were tested using ANOVAs and chi-square tests for continuous and categorical variables, respectively.

Because of the large sample size in comparisons between the No Diabetes and T1DM patients (n = 67,000), statistical comparisons for those groups may result in small *p* values even in spite of small standardized differences between the groups. Accordingly, the magnitude of differences between the groups in those analyses were further determined by calculating effect sizes (ES) using Cohen’s *d* and Cramer’s *V*, for continuous and categorical variables respectively. Cohen’s *d* is calculated using the following formula

$$d=\frac{\left|{\bar{X}}_{1}-{\bar{X}}_{2}\right|}{\sqrt{\frac{\left(\sigma_{1}^{2}+\sigma_{2}^{2}\right)}{2}}}.$$

Cohen’s *d* makes it possible to judge the magnitude of a group difference in means in a manner that is not directly dependent on sample size and can be interpreted as a difference in standard deviation unites (i.e., a Cohen’s *d* of 1 means that there is a 1 standard deviation difference in the means of the two examined groups). For this study we judge the magnitude of these differences using the standard criteria proposed by Cohen: trivial (<0.20), small (0.20 < 0.50), moderate (0.50 < 0.80) and large (≥ 0.80). Cramer’s *V* is also an effect size measure and it is used for nominal or categorical variables. Cramer’s *V* can be calculated using the following formula

$$V=\sqrt{\frac{\chi^{2}}{n(k-1)}}$$

where k represents the number of possible values of each variable. Cramer’s *V* can be interpreted in the same way as a correlation coefficient.

## Results

### Burden of T1DM

Table 1 contains the demographic characteristics collected in the NHWS split by diabetes status (T1DM vs. No Diabetes). As can be seen in the table, there were a few statistically significant differences between the groups with regard to demographics. Specifically, a higher proportion of T1DM patients were male and current or former smokers relative to patients without diabetes. Moreover, T1DM patients reported lower incomes, lower overall BMI scores, and higher Charlson Comorbidity Index (CCI)[21] scores than No Diabetes patients. All other demographic variables did not differ significantly between these groups.

**Table 1:** Demographic Differences between T1DM/No Diabetes groups.

		No Diabetes (n = 57912)		T1DM(n = 402)			
		%/M	n/SD	%/M	n/SD	*p*	*d*/*V*
Age						.160	0.01
	18 to <25	**7.9%**	4591	**6.2%**	25		
	25 to <35	**20.9%**	12117	**20.6%**	83		
	35 to <45	**19.6%**	11345	**19.7%**	79		
	45 to <55	**20.0%**	11573	**24.4%**	98		
	55 to <65	**14.5%**	8374	**11.4%**	46		
	65 and older	**17.1%**	9912	**17.7%**	71		
Sex						< .001	0.03
	Female	**55.0%**	31853	**39.8%**	160		
	Male	**45.0%**	26059	**60.2%**	242		
Employed						.625	0.00
	No	**43.3%**	25085	**44.5%**	179		
	Yes	**56.7%**	32827	**55.5%**	223		
Married/Living with Partner						.555	0.00
	No	**37.9%**	21932	**39.3%**	158		
	Yes	**62.1%**	35980	**60.7%**	244		
University Degree						.635	0.00
	No	**60.1%**	34815	**59.0%**	237		
	Yes	**39.9%**	23097	**41.0%**	165		
Income (Euros)						.047	0.01
	<20k	**25.6%**	14812	**28.1%**	113		
	20k to 50k	**44.9%**	25975	**40.3%**	162		
	50k+	**15.9%**	9221	**19.9%**	80		
	Decline	**13.6%**	7904	**11.7%**	47		
BMI (average)*		**25.77**	5.32	**24.89**	5.19	< .001	0.16
BMI category						< .001	0.02
	Underweight	**3.2%**	1831	**2.2%**	9		
	normal weight	**45.8%**	26548	**58.0%**	233		
	Overweight	**32.2%**	18651	**30.6%**	123		
	Obese	**15.9%**	9213	**7.5%**	30		
	Unknown	**2.9%**	1669	**1.7%**	7		
Weight (KG)*		**74.74**	17.33	**74.72**	17.92	.985	0.00
Smoking Status						.020	0.01
	Current	**25.3%**	14650	**29.1%**	117		
	Former	**30.2%**	17512	**33.3%**	134		
	Never	**44.5%**	25750	**37.6%**	151		
Currently Drink Alcohol						.063	0.01
	No	**22.0%**	12750	**25.9%**	104		
	Yes	**78.0%**	45162	**74.1%**	298		
Exercise (past month)						.588	0.00
	0 times	**39.6%**	22953	**38.3%**	154		
	1+ times	**60.4%**	34959	**61.7%**	248		
CCI						< .001	0.04
	0	**86.2%**	49942	**72.6%**	292		
	1	**9.5%**	5475	**15.2%**	61		
	2	**2.9%**	1687	**7.5%**	30		
	3+	**1.4%**	808	**4.7%**	19		
Time Since Diagnosis (Years)		**-**	-	**21.72**	14.69		-

d = Cohen’s d; V = Cramer’s V

*Continuous variable, reported effect size estimate is Cohen’s *d*

Table 2 contains the most common comorbidities reported among T1DM patients and the prevalence of these conditions among the No Diabetes patients. Pain, Hypertension, High Cholesterol, Depression, and COPD were all more commonly reported by T1DM patients than by No Diabetes patients. Although common among T1DM patients, rates of arthritis, nasal allergies, and asthma were not significantly more prevalent in the T1DM patients than in the No Diabetes patients.

**Table 2:** Chronic Comorbidities by T1DM/No Diabetes groups.

	No Diabetes (n = 57912)		T1DM (n = 402)			
	%	n	%	n	*p*	*V*
Pain	**22.2%**	12869	**37.8%**	152	< .001	.03
Hypertension	**16.3%**	9434	**28.1%**	113	< .001	.03
High Cholesterol	**14.0%**	8102	**25.6%**	103	< .001	.03
Depression	**10.5%**	6052	**16.9%**	68	< .001	.02
Arthritis	**9.1%**	5268	**10.9%**	44	.199	.01
Nasal Allergies	**7.1%**	4105	**7.2%**	29	.922	.00
Asthma	**6.3%**	3645	**7.7%**	31	.244	.01
COPD	**1.3%**	771	**3.0%**	12	.004	.00

Table 3 contains HRQL domains, summary scores, and health utility values by Diabetes status. With regard to HRQL, T1DM patients reported lower scores than No Diabetes patients across all HRQL measures examined. The largest differences were for the general health domain score and the Physical component summary score. It is worth noting that the largest impairment seems to be physical in nature as the difference between the two groups was roughly twice as large for PCS scores relative to MCS scores.

**Table 3:** Health status by Type 1 Diabetes Status.

	No Diabetes (n = 57912)		T1DM (n = 402)			
SF-36	M	SD	M	SD	*p*	d*
Bodily Pain	**49.63**	10.27	**46.35**	11.03	< .001	.31
General Health	**49.34**	9.58	**40.92**	10.20	< .001	.85
Mental Health	**45.96**	10.39	**43.66**	11.31	< .001	.21
Physical Functioning	**51.68**	8.41	**47.88**	10.11	< .001	.41
Role Emotional	**47.88**	10.57	**44.33**	12.03	< .001	.31
Role Physical	**49.74**	9.21	**44.98**	10.79	< .001	.47
Social Functioning	**48.13**	9.85	**44.64**	11.14	< .001	.33
Vitality	**49.46**	9.32	**46.05**	9.71	< .001	.35
Physical Component Summary	**51.63**	8.70	**46.33**	9.67	< .001	.57
Mental Component Summary	**46.18**	10.60	**43.70**	11.20	< .001	.22
Health Utilities	**0.72**	0.13	**0.67**	0.14	< .001	.37

*Cohen’s d = <0.20 = trivial; 0.20 < 0.50 = small; 0.50 < 0.80 = moderate; ≥ 0.80 = large

T1DM patients also reported significantly greater presenteeism, overall work productivity impairment, and activity impairment than No Diabetes patients (Table 4). However, it should be noted that work productivity measures were only assessed among those currently employed, thus these results include smaller sample sizes than other measure such as activity imapriment, HRQL and resource use. There were no significant differences in absenteeism between T1DM patients and No Diabetes patients (*p* = .154). These results, taken together with the HRQL results, suggest that T1DM is a disabling condition–particularly with regard to daily physical activities outside of work.

**Table 4:** Work Productivity Loss, Activity Impairment, and Healthcare Resource Use by T1DM/No Diabetes groups.

	No Diabetes (n = 57912)		T1DM (n = 402)			
	M	SD	M	SD	*p*	d**
Work Productivity and Activity Impairment*						
Absenteeism (%)	**5.45**	18.51	**7.22**	18.04	.154	0.10
Presenteeism (%)	**16.35**	23.69	**27.45**	27.94	< .001	0.47
Work Productivity Loss (%)	**19.74**	28.05	**30.91**	30.95	< .001	0.40
Activity Impairment (%)	**23.95**	28.15	**36.29**	30.89	< .001	0.44
*Healthcare Resource Use*						
ER Visits (#)	**0.19**	0.99	**0.38**	0.96	< .001	0.19
Hospitalizations (#)	**0.12**	0.77	**0.30**	1.10	.001	0.23
HCP Visits (#)	**4.43**	6.20	**7.84**	9.37	< .001	0.55

*Note sample sizes differ for the WPAI because absenteeism (T1DM n = 223, No Diabetes n = 32827), presenteeism (T1DM n = 220, No Diabetes n = 32163), and work productivity loss (T1DM n = 223, No Diabetes n = 32827) are only assessed for those who are currently employed. Activity Impairment was assessed for all participants regardless of employment status.

**Cohen’s d = <0.20 = trivial; 0.20 < 0.50 = small; 0.50 < 0.80 = moderate; ≥ 0.80 = large

Finally, T1DM patients reported more ER visits, hospitalizations, and HCP visits in the past 6 months than No Diabetes patients (Table 4). These results suggest that T1DM patients use more resources for both preventative and reactive healthcare.

### Diabetes Complications

The impact of the number of complications in T1DM patients on the burden of illness was examined by splitting the T1DM patients according to their total number of reported complications (None, One, Two or more). Demographic characteristics by these groups are reported in Table 5. There were a few statistically significant differences worth noting. First, patients with two or more complications had a significantly higher mean BMI score than those with one or none. Consequently, patients with two or more complications also had a higher mean body weight than those with one or none. Fewer patients with two or more complications reported having exercised in the past month than those with one or no complications. However, this difference was only statistically significant between the two or more complications and no complications groups. A similar pattern was observed for Charlson Comorbidity Index (CCI) scores in that a higher proportion of the patients with two or more complications reported a CCI score of > 0 than patients with one or no complications. Patients with two or more complications also reported a significantly longer time since diagnosis relative to those with no complications.

**Table 5:** Demographic differences by T1DM with and without complications.

		Type 1 Diabetes by Complications						
		None (n = 270)		One (n = 76)		Two or more (n = 56)		
		%/M	n/SD	%/M	n/SD	%/M	n/SD	*p*
Age								.174
	18 to <25	**7.8%**	21_a_	**1.3%**	1_a_	**5.4%**	3_a_	
	25 to <35	**23.7%**	64_a_	**17.1%**	13_a_	**10.7%**	6_a_	
	35 to <45	**19.3%**	52_a_	**19.7%**	15_a_	**21.4%**	12_a_	
	45 to <55	**21.9%**	59_a_	**28.9%**	22_a_	**30.4%**	17_a_	
	55 to <65	**10.0%**	27_a_	**17.1%**	13_a_	**10.7%**	6_a_	
	65 and older	**17.4%**	47_a_	**15.8%**	12_a_	**21.4%**	12_a_	
Sex								.659
	Female	**40.4%**	109_a_	**35.5%**	27_a_	**42.9%**	24_a_	
	Male	**59.6%**	161_a_	**64.5%**	49_a_	**57.1%**	32_a_	
Employed								.212
	No	**42.6%**	115	**43.4%**	33	**55.4%**	179	
	Yes	**57.4%**	155	**56.6%**	43	**44.6%**	223	
Married/Living with Partner								.669
	No	**40.0%**	108_a_	**40.8%**	31_a_	**33.9%**	19_a_	
	Yes	**60.0%**	162_a_	**59.2%**	45_a_	**66.1%**	37_a_	
University Degree								.926
	No	**58.9%**	159_a_	**60.5%**	46_a_	**57.1%**	32_a_	
	Yes	**41.1%**	111_a_	**39.5%**	30_a_	**42.9%**	24_a_	
Income (Euros)								.538
	<20k	**25.6%**	69_a_	**34.2%**	26_a_	**32.1%**	18_a_	
	20k to 50k	**41.1%**	111_a_	**38.2%**	29_a_	**39.3%**	22_a_	
	50k+	**19.6%**	53_a_	**19.7%**	15_a_	**21.4%**	12_a_	
	Decline	**13.7%**	37_a_	**7.9%**	6_a_	**7.1%**	4_a_	
BMI (average)		**24.52**_a_	4.04	**24.58**_a_	3.40	**27.04**_b_	9.69	.004
BMI category								.036
	underweight	**1.9%**	5_a_	**2.6%**	2_a_	**3.6%**	2_a_	
	normal weight	**59.6%**	161_a_	**56.6%**	43_a_	**51.8%**	29_a_	
	overweight	**30.7%**	83_a_	**34.2%**	26_a_	**25.0%**	14_a_	
	obese	**5.6%**	15_a_	**5.3%**	4_a_	**19.6%**	11_b_	
	unknown	**2.2%**	6_a_	**1.3%**	1_a_	**0.0%**	0^1^	
Weight (KG)		**73.60**_**a**_	14.89	**73.48**_**a**_	14.29	**81.70**_**b**_	30.13	.007
Smoking Status								.075
	Current	**29.3%**	79_a_	**31.6%**	24_a_	**25.0%**	14_a_	
	Former	**29.6%**	80_a_	**35.5%**	27_a,b_	**48.2%**	27_b_	
	Never	**41.1%**	111_a_	**32.9%**	25_a_	**26.8%**	15_a_	
Currently Drink Alcohol								.015
	No	**29.6%**	80_a_	**13.2%**	10_b_	**25.0%**	14_a,b_	
	Yes	**70.4%**	190_a_	**86.8%**	66_b_	**75.0%**	42_a,b_	
Exercise (past month)								.006
	0 times	**34.4%**	93_a_	**38.2%**	29_a,b_	**57.1%**	32_b_	
	1+ times	**65.6%**	177_a_	**61.8%**	47_a,b_	**42.9%**	24_b_	
CCI								< .001
	0	**79.6%**	215_a_	**73.7%**	56_a_	**37.5%**	21_b_	
	1	**11.1%**	30_a_	**14.5%**	11_a_	**35.7%**	20_b_	
	2	**6.3%**	17_a_	**7.9%**	6_a_	**12.5%**	7_a_	
	3+	**3.0%**	8_a_	**3.9%**	3_a,b_	**14.3%**	8_b_	
	Time Since Diagnosis (Years)	**19.37**_a_	14.02	**24.55**_b_	14.47	**29.29**_b_	15.23	< .001

Note: Values in the same row and subtable not sharing the same subscript are significantly different at p< .05 in the two-sided test of equality for column proportions. Cells with no subscript are not included in the test. Tests assume equal variances. Tests are adjusted for all pairwise comparisons within a row of each innermost subtable using the Bonferroni correction.

Tables 6 and 7 contain bivariates comparing health outcomes by number of complications. Overall ANOVAs were statistically significant for all measures examined with the exception of absenteeism (*p* = .059). In general, the higher the number of complications, the lower the patient rated his or her health status with regard to the SF-36 domain scores, MCS, PCS and health utilities. However, not all pairwise comparisons were statistically significant. Similarly as the number of complications increases from none to one to two or more, estimates of presenteeism, work productivity loss, and activity impairment also increase. However, the differences between one and two or more complications were not statistically significant for these comparisons. Again, measures of presenteeism and absenteeism are limited to only those respondents who were employed at the time of the survey and thus are subject to smaller base sample size than all other metrics examined. Finally, estimates of healthcare resource utilization increase as the number of complications increase. The largest differences were for the two or more complications group whose resource use was roughly double that of those with one complication across all healthcare resource use metrics.

**Table 6:** Health status by Type 1 Diabetes Complication Status.

	None (n = 270)		One (n = 76)		Two or more (n = 56)		
	M	SD	M	SD	M	SD	*p*
Bodily Pain	**48.26**_**a**_	10.86	**44.20**_**b**_	10.50	**40.10**_**c**_	9.79	< .001
General Health	**42.70**_**a**_	9.96	**39.33**_**b**_	9.02	**34.54**_**c**_	10.09	< .001
Mental Health	**44.85**_**a**_	11.23	**41.95**_**b**_	10.62	**40.26**_**b**_	11.81	.007
Physical Functioning	**49.99**_**a**_	9.12	**45.78**_**b**_	9.44	**40.52**_**c**_	11.57	< .001
Role Emotional	**45.89**_**a**_	11.39	**43.11**_**a**_	11.36	**38.45**_**b**_	13.96	< .001
Role Physical	**47.10**_**a**_	9.88	**42.09**_**b**_	11.10	**38.67**_**b**_	11.37	< .001
Social Functioning	**45.94**_**a**_	11.04	**43.42**_**a,b**_	9.95	**40.06**_**b**_	11.94	.001
Vitality	**47.05**_**a**_	9.71	**45.41**_**a,b**_	8.18	**42.09**_**b**_	10.61	.002
Physical Component Summary	**44.57**_**a**_	11.28	**42.93**_**a,b**_	9.79	**40.51**_**b**_	12.10	.037
Mental Component Summary	**48.47**_**a**_	8.91	**43.95**_**b**_	8.93	**39.22**_**c**_	10.13	< .001
Health Utilities	**.69**_**a**_	.14	**.63**_**b**_	.12	**.60**_**b**_	.14	< .001

Note: Values in the same row and subtable not sharing the same subscript are significantly different at p< .05 in the two-sided test of equality for column means. Cells with no subscript are not included in the test. Tests assume equal variances.

**Table 7:** Demographic differences by T1DM with and without complications.

		Type 1 Diabetes by Complications						
		None (n = 270)		One (n = 76)		Two or more (n = 56)		
		M	SD	M	SD	M	SD	*p*
Work Productivity and Activity Impairment*								
	Absenteeism	**6.10**_**a**_	18.51	**6.58**_**a**_	11.62	**15.28**_**a**_	22.34	.059
	Presenteeism	**21.51**_**a**_	25.60	**38.60**_**b**_	27.31	**44.40**_**b**_	30.83	< .001
	Work Productivity Loss	**24.71**_**a**_	29.34	**41.53**_**b**_	28.20	**51.08**_**b**_	32.72	< .001
	Activity Impairment	**30.56**_**a**_	29.05	**43.82**_**b**_	29.26	**53.75**_**b**_	33.33	< .001
*Healthcare Resource Use*								
	ER Visits	**0.29**_**a**_	0.79	**0.42**_**a,b**_	0.93	**0.73**_**b**_	1.52	.006
	Hospitalizations	**0.24**_**a**_	1.07	**0.25**_**a,b**_	0.52	**0.66**_**b**_	1.64	.032
	HCP Visits	**6.28**_**a**_	7.45	**7.96**_**a**_	7.65	**15.20**_**b**_	14.87	< .001

*Note sample sizes differ for the WPAI because absenteeism (None n = 155, One n = 43, Two or more n = 25), presenteeism (None n = 152, One n = 43, Two or more n = 25), and work productivity loss (None n = 155, One n = 43, Two or more n = 25) are only assessed for those who are currently employed. Activity Impairment was assessed for all participants regardless of employment status.

## Discussion

### Findings

This study surveyed a large sample of European adults with self-reported T1DM diagnosis to provide an updated and comprehensive review on the impact of T1DM diagnosis and its complications on humanistic outcomes. In line with the previously described bodies of literature,[9,10,12,13] we found T1DM patients to have poorer health, and the diagnosis to be significantly associated with poorer HRQL and (likely-related) heightened work impairment and medical resource use. These poor outcomes were exacerbated by the presence of T1DM complications, especially as the number of complications increased.

The average T1DM patient in our study was male, middle-aged, and married, with a high school-level education. The T1DM patients tended to be of normal weight, with a history of smoking (either current or former), and were reportedly current alcohol-users. The higher proportion of smoking and alcohol use in the T1DM patients is disconcerting in view of the various guideline recommendations strongly encouraging smoking cessation and the restrictions on alcohol use in T1DM patients. Compared to control subjects, T1DM patients reported significantly higher prevalence of pain conditions, hypertension, hypercholesterolemia, depressive symptomatology, and COPD. T1DM patients with the greatest number of complications (i.e., ≥ 2) had significantly higher BMI, exercised significantly less often, and had more medical comorbidities relative to patients with only one complication or no complications. These findings are consistent with those from previous studies of European T1DM patients, [14,15] although the current study reports on a wider array of complications.

In this study T1DM patients showed significantly lower HRQL than No Diabetes patients across domains, which is significant when considered in light of past research. For instance, a study by Bjørner et al. interpreted score differences in the SF-36 vitality domain in patients with different chronic conditions, including diabetes and found that patients suffering from a condition and had a 5-point lower vitality score (compared with patients without that condition) had significantly increased odds of inability to work (odds ratio, OR, 1.27), job loss within 1 year (OR 1.13) and hospitalization within 1 year (OR 1.08). Patients with diabetes had especially high OR for hospitalization (OR 1.63).[22] A more recent analysis found that a decrement of 1 point on the physical function, general health, and physical component summary scores of the SF 36 conferred a relative risk of mortality of 1.05 to 1.09.[23] Thus, decrements in HRQoL associated with T1DM can signal potential issues related to hospitalization, work productivity loss, and indeed, mortality.

Moreover, the presence and number of T1DM complications appears to compound these difficulties. In this study, T1DM patients with two or more complications showed the lowest HRQL across all subscales, the poorest work productivity across all subscales (excepting absenteeism), and the greatest use of medical resources compared with patients with one or no complications. Similarly, patients with one complication reported significantly poorer outcomes across these measures relative to patients with no complications. Presenteeism and work productivity loss averages were twice as high among patients with ≥ 2 complications relative to patients with no complications. The average number of emergency room visits, number of hospitalizations, and number of primary care provider visits were also twice as high among patients with ≥ 2 complications compared to patients with no complications. As the T1DM group reported poorer health and lowered HRQL, these findings make clinical sense. Nonetheless, this is the first study we are aware of to extend research of the number of T1DM complications to direct and indirect economic burden in Europe.

### Limitations

Limitations are inherent to studies with cross-sectional, self-reported data, which we acknowledge here. Study data, such as T1DM diagnosis and number of complications, are self-reported and retrospective and thus cannot be clinically verified. Lost work productivity and medical visits were also self-reported, and recall bias may have resulted in over- or under-estimation. Relatedly, patients who self-reported a diagnosis of diabetes were given the choice of “type 1 or juvenile diabetes” or “type 2 or adult onset diabetes” to identify their diagnosis. Accordingly, it is possible that some patients who were diagnosed with type 1 diabetes as adults erroneously selected “adult onset” when answering this question. Second, the data are cross-sectional and as such, no claims about cause and effect are possible. Third, as T1DM is a disease with extremely complex etiologies and medical sequelae, additional unmeasured variables may have impacted our results. Fourth, we did not adjust for multiple comparisons in these analyses, however many of the p values were so small to begin with because of the relatively large sample size that this likely would not have affected the results. In an attempt to address this limitation we also provided Cohen’s *d* estimates for analyses with small sample sizes and instead focused on the magnitude of the effect rather than its presence or absence. These small sample sizes also prevented us from having large enough samples to complete meaningful country-specific comparisons. Finally, the reasons as to why fewer individuals in T1DM group were obese relative to controls remain unknown, as T1DM patients tend to be overweight [24]. However, this may be related to group sociodemographic differences (e.g., gender, which has been shown to relate to BMI in studies comparing T1DM with T2DM and control patients) [25] and/or unmeasured factors related to research participation willingness.

## Conclusions

Study results indicate that a diagnosis of T1DM decreases health status and impacts both direct and indirect costs significantly. Moreover, this burden increases with a greater number of T1DM complications. Although there have been advances in the treatment of T1DM and its associated conditions, the degree of disease-related patient suffering, work productivity loss, and health care system burden appears to remain substantial. Weight loss in European T2DM patients has been shown to improve HRQL and work productivity and decrease medical resource use, so it may be useful in T1DM patients, but has yet to be proven.[26] Ultimately, prevention and improved management of T1DM in European patients is needed.
